# Paternity seven years after a negative post-vasectomy semen analysis: a case report

**DOI:** 10.1186/s13256-020-02374-0

**Published:** 2020-04-22

**Authors:** Athena Michaelides, Mehrban Ghani

**Affiliations:** 1 University of Nicosia Medical School, Nicosia, Cyprus; 2 White Rose Medical Practice, Cudworth, UK

**Keywords:** Vasectomy, Recanalization, Paternity, Semen analysis, Conception

## Abstract

**Background:**

Vasectomy is one of the permanent methods of contraception; however, the risk of conception still exists. Early failure, defined as a postoperative semen analysis showing persistent motile sperm, occurs in 1 in every 250 patients. Late failure, defined as the rejoining of the severed ends of the vas deferens, occurs in 1 in every 2000 patients.

**Case presentation:**

A healthy 37-year-old British male presented to our clinic because his wife had conceived seven years after he had undergone a vasectomy. The result of his semen analysis after the vasectomy was negative, and the histopathological report confirmed that the sample contained tissue from both the left and right vas deferens. His wife conceived seven years after the procedure, and semen analysis at that time confirmed a total of 0.5 million sperm per milliliter of semen in a total semen sample of 6.3 ml. The total number of motile sperm recorded was 2.5 million.

**Conclusion:**

This case shows that late recanalization can occur up to seven years after a vasectomy. Patients should be informed prior to the procedure that late recanalization, although rare, may still occur. Post-vasectomy paternity necessitates further counseling to ensure that the patient and the patient’s partner fully understand the implications and options available to them.

## Background

Vasectomy is recognized as one of the permanent methods of contraception; however, the risk of conception after vasectomy still exists [1–3]. Vasectomy failure can be due to technical error during the procedure, early recanalization, late recanalization, or patients’ failing to use alternative contraceptive methods immediately after the procedure when sperm could still be present in the ejaculate [4].

There is a general consensus that men are considered sterile if < 100,000 non-motile spermatozoa per milliliter are present in the ejaculate sample taken three months after the vasectomy [4, 5]. Early failure, defined as a postoperative semen analysis showing persistent motile sperm, occurs in 1 in every 250 patients. Late failure, defined as the rejoining of the severed ends of the vas deferens, occurs in 1 in every 2000 patients [6]. This report aims to describe the case of a young man who became a father after a negative post-vasectomy semen analysis.

## Case presentation

A healthy 37-year-old British male came to our clinic because his wife had conceived seven years after he had undergone a vasectomy. The decision to have a vasectomy was motivated by him already having two children. The result of his semen analysis post-vasectomy was negative: no sperm were detected in a 5 ml sample of his semen. The histopathological report confirmed that the vasectomy sample contained a strip of tissue 1.5 cm in length from both the left and right vas deferens. This confirmed that the procedure was carried out successfully, and paternity seven years later could not be attributed to technical failure.

Seven years after the procedure, the patient’s wife conceived. Semen analysis at this time confirmed that sperm were present within the sample. A total of 0.5 million sperm per milliliter of semen (World Health Organization [WHO] normal reference range, > 15 million/ml) was recorded in a total semen volume of 6.3 ml. The total number of progressive motile sperm recorded was 2.5 million (WHO normal reference range, > 7.2 million). This case shows that late recanalization can occur up to seven years after a vasectomy and despite oligospermia, conception is still possible.

## Discussion and conclusions

Vasectomy is one of the safest methods of male contraception. Voluntary vasectomy is available to men of any age and family size as part of advanced family planning [7]. A preoperative discussion between the physician and the patient includes the consideration of the patient’s age, his current partnership status, and his irrevocable decision to undergo a procedure with a permanent outcome of sterility. The risks and complications are also discussed [7].

The procedure is usually carried out in an outpatient setting with the patient under local anesthesia, but general anesthesia may be used in some instances. Different vasectomy techiques may be employed. The standard ligation technique consists of identification and resection of 2–4 cm of the vas deferens. The fulguration technique employs a diathermic needle to create thermal damage to the mucosa of the vas by inducing an inflammatory reaction that results in occlusion. The “no-scalpel” vasectomy employs a clamp to perforate and separate the skin covering the vas. The vas is then grasped with forceps to produce a result similar to that of the fulguration technique [7].

The more common complications of a vasectomy include mild scrotal swelling and bruising, bleeding and postoperative hematoma formation, blood in the semen of the first few postprocedural ejaculations, chronic testicular pain that may affect day-to-day activities, and epididymo-orchitis or testicular infection and inflammation [6, 8]. The less common complications are early and late failure, which occur in 1 in 250 patients and 1 in 2000 patients, respectively [6].

Studies have shown that early recanalization usually occurs between 2 and 6 weeks post-vasectomy [9, 10]. Our present case report describes failure due to late recanalization that occurred seven years post-vasectomy. Recanalization occurs when epithelial microtubules proliferate through the granulomatous tissue between the severed ends of the vas deferens producing a fistula that allows sperm to pass through [10]. Recanalization is more likely to occur if less than 1 cm of the vas is removed during the surgery or if an abscess forms after the procedure [3]. Because our patient’s histopathological report confirmed 1.5 cm of vas removed from each testis, our patient does not fall into the group at high risk of recanalization.

Vasectomy, although mostly successful, still has a degree of failure. Patients should be counseled prior to the procedure that late recanalization may occur in 1 in every 2000 cases. Post-vasectomy paternity necessitates further counseling to ensure that the patient and the patient’s partner fully understand the implications and options available to them.

## Data Availability

The patient’s information and medical records used for the case report are available from the corresponding author upon request.

## Abbreviation

WHO: World Health Organization
